# Direct Synthesis of Multicolor Fluorescent Hollow Carbon Spheres Encapsulating Enriched Carbon Dots

**DOI:** 10.1038/srep19382

**Published:** 2016-01-25

**Authors:** Qiao-Ling Chen, Wen-Qing Ji, Su Chen

**Affiliations:** 1 State Key Laboratory of Materials-Oriented Chemical Engineering and College of Chemistry and Chemical Engineering, Nanjing Tech University (former: Nanjing University of Technology), Nanjing 210009 (P. R. China)

## Abstract

Multicolor fluorescent hollow carbon spheres (HCSs) are fabricated by an easy one-step route of *in situ* pyrolysis process with the use of natural scales and collagen powders as the precursor. The gas blow forming mechanism and photoluminescence (PL) emission mechanism of HCSs have been thoroughly discussed and proved that HCSs represent the first examples of three-dimensional multicolor fluorescent nanomaterials based on carbon dots (CDs). The HCSs encapsulate enriched carbon dots with high quantum yields (QYs) of 38%, and thus are applied in inkjet printing and sensitized solar cells. This strategy offers a promising avenue for preparing multicolor fluorescent hollow carbon materials on an industrial scale.

The discoveries of carbon nanotubes, fullerene, graphene and close spherical carbon shell as new kinds of materials have opened an emerging field of interdisciplinary science across a broad spectrum of disciplines. In recent decades, hollow carbon spheres (HCSs) have inspired great interests owing to their potentials in fundamental research and industrial applications such as electrode materials^1^,^2^,^3^, catalysis supports^4^,^5^,^6^,^7^, supercapacitors^8^, and hydrogen storage^9^. Till now, various approaches have been developed for the synthesis of HCSs with low density, high specific surface area, good chemical stability, and available hollow interiors^10^, by using many catalytic methods including template synthesis^11^,^12^,^13^, solvothermal pyrolysis^14^, microwave preparation^15^,^16^ and direct chemical reaction^17^,^18^. In this respect, Mokaya and co-workers synthesized mesoporous carbon hollow spheres using mesoporous silica as template, and subsequently etched silica template to produce HCSs^13^. Wu and co-workers prepared hollow carbon nanospheres using Fe(C_5_H_5_)_2_ and C_5_Cl_6_ as starting materials^8^. Liu and co-workers developed a simple *in situ* template method to fabricate carbon hollow structures by the controlled thermolysis of Zn(Ac)_2_ in the presence of ethanol, followed by removal of ZnO cores with HCl etching^14^. However, despite amazing achievements that have been made in HCS synthesis, most of the previously reported methods often involve complicated multi-step, energy-consuming, and time-consuming procedures or harsh post treating conditions, and highly rely on expensive or poisonous precursors and catalysts, thereby limiting facile production of HCSs on a large scale and their industrial application. In addition, the fabrication of fluorescent HCSs has not been described yet, although several literatures have reported fluorescent carbon-based materials, such as carbon nanotubes fragments^19^,^20^,^21^, graphene quantum dots^22^,^23^, nanodiamonds^24^,^25^ and carbon dots (CDs)^26^,^27^. The breakthrough of synthesis of HCSs on a large scale, along with advanced functional properties, is therefore highly expected.

Herein, we present an alternative facile method for production of HCSs with multicolor fluorescence *via in situ* pyrolysis of fish scales for the first time. To the best of our knowledge, *in situ* synthesis of HCSs is rare, and HCSs with versatile fluorescent properties have rarely been reported before. Furthermore, we develop a new avenue for production of multicolor fluorescent HCSs on a large scale *via* simple pyrolysis method, only using collagen powder tablets as starting materials. Collagen is inexpensive and rich material, which is also a main component of fish scales. Therefore, this method is quite general, allows the formation of HCSs to be carried out in an easy way and on a large scale, and might open up a new route to develop hollow carbon materials.

## Results and Discussion

**Figure 1:**
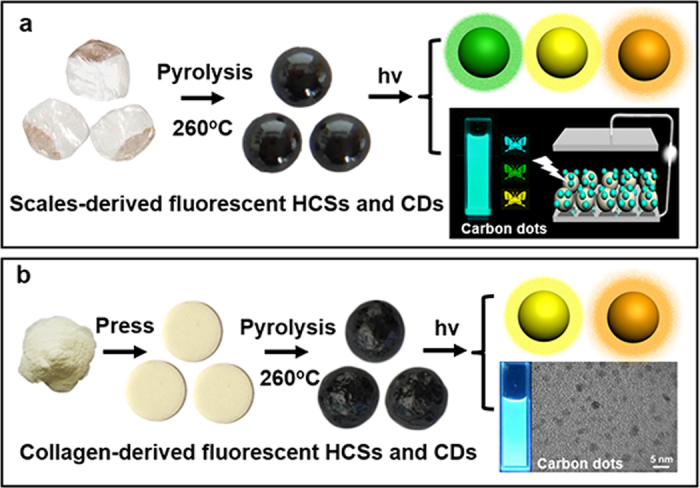
(**a**) Schematic synthesis of multicolor fluorescent HCSs *via in situ* pyrolysis from fish scales and their applications in inkjet printing and sensitized solar cells. (**b**) A new route of preparing HCSs from collagen powders on a large scale.

The presented approach for the synthesis of fluorescent HCSs is simple, involving one-step pyrolysis process (Fig. 1). Initially, we chose pretreated whitefish scales as the starting materials, and then carbonized them at 260 °C for 2 h under an atmosphere of purified nitrogen, to obtain HCSs with multicolor fluorescence (Fig. 1a). In addition, these HCSs could further produce CDs by simply crushing down to nanoscale. The as-prepared CDs display bright cyan fluorescence under UV lamp (365 nm) and disperse well into organic solvents. This result suggests that the multicolor fluorescent properties of HCSs should be originated from a number of fluorescent CDs distributed onto the surface of HCSs. The applications of CDs as the fluorescent ink of inkjet printing and the sensitizer of solar cell were also demonstrated (Fig. 1a). On the other hand, inspired by this strategy, we further tried to explore an easier one-step method to produce multicolor fluorescent HCSs from collagen powders than from the scales, because the resource of whitefish scale is limited and the pretreatment of scale is time-consuming. Then collagen powders were employed as the precursor, pressed into tablets, and treated with thermal pyrolysis at 260 °C for 2 h. As expected, HCSs with multicolor fluorescence also grew up, suggesting that HCSs could be facilely produced on an industrial scale *via* pyrolysis of collagen powder tablets, without any pretreatments (Fig. 1b). Still, these HCSs could be crushed down to nanoscale to form CDs.

### Forming mechanism of HCSs

**Figure 2:**
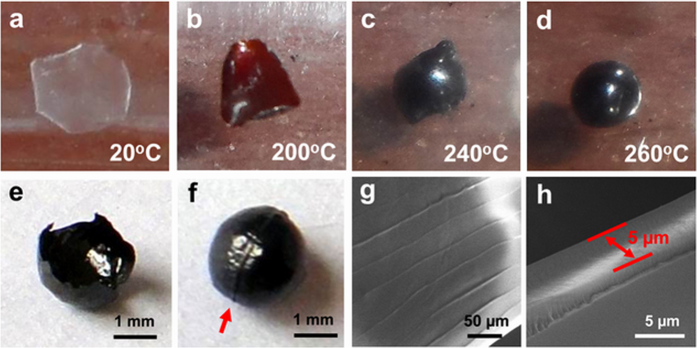
(**a–d**) Photographs of a HCS formation in various stages of pyrolysis. (**e**) Photograph of a broken HCS. (**f**) Photograph of a HCS’s waist. (**g**) SEM image of the outer surface of a HCS. (**h**) SEM image of the wall thickness of a HCS.

Pyrolysis process of fish scales at various stages were carefully observed, and photographed (Fig. 2). The shape of the whitefish scales is irregular and mostly approximately round with diameters about 2–5 mm (Fig. 2a). As reported, the scales have a structure of stratified lamellae, consisting of external osseous layers and internal dense collagen lamellae^28^. The external osseous layers have been already removed during the pretreatment process, and the internal collagen lamellae are left. As the furnace temperature increased to 200 °C, the transparent scale piece turned brown and curled (Fig. 2b). When the temperature exceeded 240 °C, the scale was gradually bulging from its center (Fig. 2c), and pungent gas was given off, simultaneously. The inner gas originated from collagen’s pyrolysis couldn’t escape due to the dense surface of the scale, which makes the upper surface of the scale arch upward and lower surface arch downward. This process is similar to the gas blow forming. Finally, a dense, smooth, black and totally enclosed sphere was forming with diameters of 1–3 mm under pyrolysis at 260 °C for 2 h (Fig. 2d). As shown in Fig. 2e, the obtained light and fragile carbon sphere is hollow and the inner wall is also smooth. Otherwise, there is a boundary line on the waist of sphere (Fig. 2f). This line corresponding to the outer edge of the scale provides further evidence that the HCS grows up by gas blow forming (Fig. 2a). SEM measurements indicate that the outer surface of the HCS without any pores and cracks is quite dense and prevents the pyrolysis gas from escaping (Fig. 2g). The parallel microscale curves named as characteristic growth rings, appearing on the surface of the HCS, have the spacing of approximate 40 μm, which also exist on the surface of the precursor (Figure S1). This phenomenon reveals that HCSs still maintain their microstructure of scales after pyrolysis. The wall thickness of a HCS is around 5 μm, as seen in Fig. 2h.

**Figure 3:**
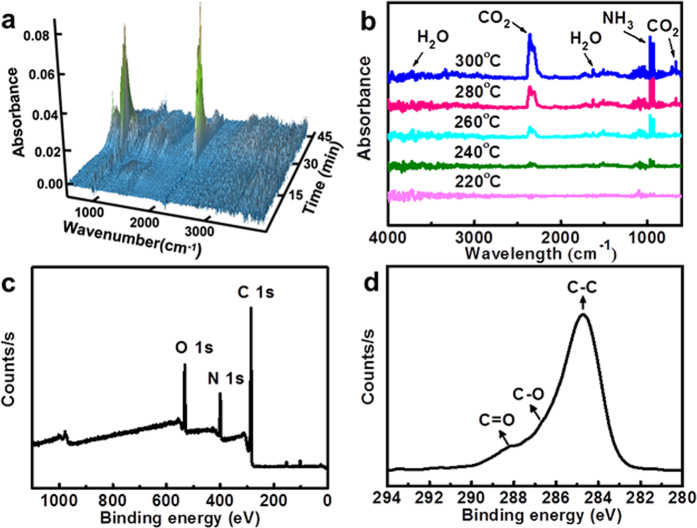
(**a**) Three-dimensional (3D) FT-IR profile (40 °C at 0 min to 500 °C at 46 min) and (**b**) the evolutionary FT-IR spectra (220 °C to 300 °C) of the gas produced from scales combustion. (**c**) XPS spectrum of the HCS and (**d**) the corresponding high resolution XPS spectrum of the C 1 s peak.

To clarify the formation of scale-derived HCSs, thermogravimetric analysis (TGA)/FT-IR spectra and XPS spectra were used to record the pyrolysis process of scales (Fig. 3). As shown in Fig. 3a,b, the precursor started to produce gases at *ca*. 240 °C (20 min), generating a massive amount of CO_2_ (2361 cm^−1^ and 669 cm^−1^), NH_3_ (964 cm^−1^ and 931 cm^−1^) along with less H_2_O (1512 cm^−1^, 1626 cm^−1^ and 3736 cm^−1^) gases. We noticed that the absorption intensity of these gases gradually increased from 240 °C to 300 °C and rapidly decreased from 300 °C to 500 °C, which demonstrates the forming temperature of HCSs above 240 °C. From 40 to 500 °C, the mass loss of the scale was 68.3%, as shown in Figure S2. Therefore, we consider that scales may undergo complex carbonization at this temperature-rise period.

### Chemical composition and structure of HCSs

The elemental analysis reveals the composition of the HCSs as follows: C 62.26 wt %, H 4.30 wt %, N 17.70 wt %. The Raman spectrum of HCSs shows peaks at 1590 cm^−1^(G band) and 1357 cm^−1^ (D band), revealing the presence of both sp^2^ and sp^3^ hybrid carbons (Figure S3)^29^. The intensity ratio of the D and G band (*I*_D_/*I*_G_) is a measurement of the disorder extent and the ratio of sp^3^/sp^2^ carbon. The HCSs shows a noticeable increase in *I*_D_/*I*_G_ ratio to 0.805 by comparison with the graphite that has a weak *I*_D_/*I*_G_ ratio of 0.365^30^. Thus, there are more vacant lattice sites and disordered carbon in HCSs than in graphite. As proposed in Figure S4, X-ray diffraction (XRD) pattern of the HCSs shows an (002) interlayer spacing of 3.7 Å, which is larger than that of bulk graphite (3.3 Å), indicating poor crystallization of HCSs^31^. X-ray photoelectron spectroscopy (XPS) spectra of HCSs indicate the existence of carbon (C 1 s, 285 eV), nitrogen (N 1 s, 399 eV) and oxygen (O 1 s, 532 eV) (Fig. 3c). In the high resolution of C 1 s peak (Fig. 3d), the positions at 284.6 eV, 286.6 eV and 288.2 eV demonstrate the presence of C−C, C−O, and C = O functional groups^32^, which are further confirmed by FT-IR spectra. As seen in Figure S5, the FT-IR spectrum of HCSs shows obvious peaks around 3433 cm^−1^ and 1653 cm^−1^, which can be ascribed to the stretching vibrations of O−H and C = O, respectively. In addition, the absorption peaks in the spectrum of HCSs around 1419 cm^−1^ belongs to C−N stretching vibrations, and 1093 cm^−1^ can be identified as C−O stretching vibrations. In the FT-IR spectrum of scales, the strong absorption peaks at 1646 cm^−1^, 1538 cm^−1^ and 1238 cm^−1^ represent amide-I, amide-II and amide-III characteristic peaks of collagen, respectively^33^. The comparison between IR spectra of HCSs and scales suggests the structure of the scales has been entirely decomposed during the pyrolysis process.

### PL properties of HCSs

**Figure 4:**
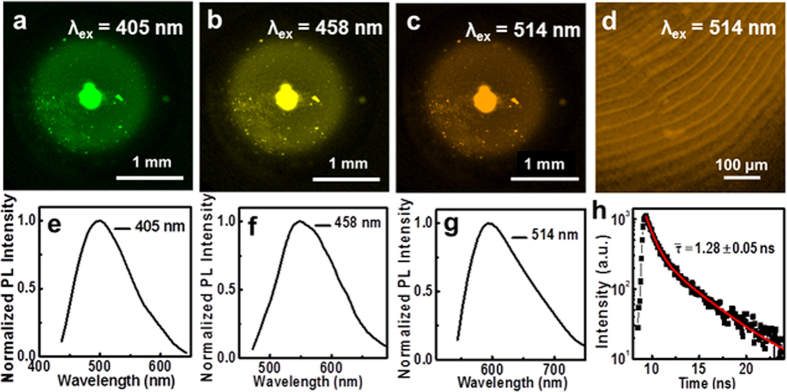
Laser confocal fluorescence microscopy (LCFM) images of a HCS at different excitation wavelengths (**a**) 405 nm, (**b**) 458 nm and (**c**) 514 nm. (**d**) is the enlargement LCFM image of a HCS (*λ*_ex_ = 514 nm). PL emission spectra of a HCS at different excitation wavelengths (**e**) 405 nm, (**f**) 458 nm and (**g**) 514 nm. (**h**) A typical time-resolved fluorescence decay curve of a HCS (*λ*_ex_ = 405 nm).

To provide insight into the PL characteristic of the HCSs, we therefore chose laser confocal fluorescence microscopy (LCFM) to examine the microstructures and fluorescence properties of HCSs. Confocal fluorescence micrographs of a HCS show that the green, yellow and orange luminescence emissions were observed with the excitation wavelength of 405 nm, 458 nm and 514 nm, respectively (Fig. 4a–c). Corresponding to Fig. 4a–c, the PL emission spectra of the HCS (Fig. 4e–g) were measured by LCFM measurements. The emission spectra of the HCS show a typical excitation-dependent feature that the PL peak shifts to longer wavelength as the excitation wavelength gradually increases. Additionally, from the enlargement LCFM image of a HCS (Fig. 4d), the HCS shows clear characteristic growth rings, which are in agreement with the SEM image of HCS (Fig. 2g). The time-resolved photoluminescence measurement indicates that the decay lifetime of HCSs is 1.28 ± 0.05 ns (Fig. 4h).

### PL emission mechanism of HCSs

**Figure 5:**
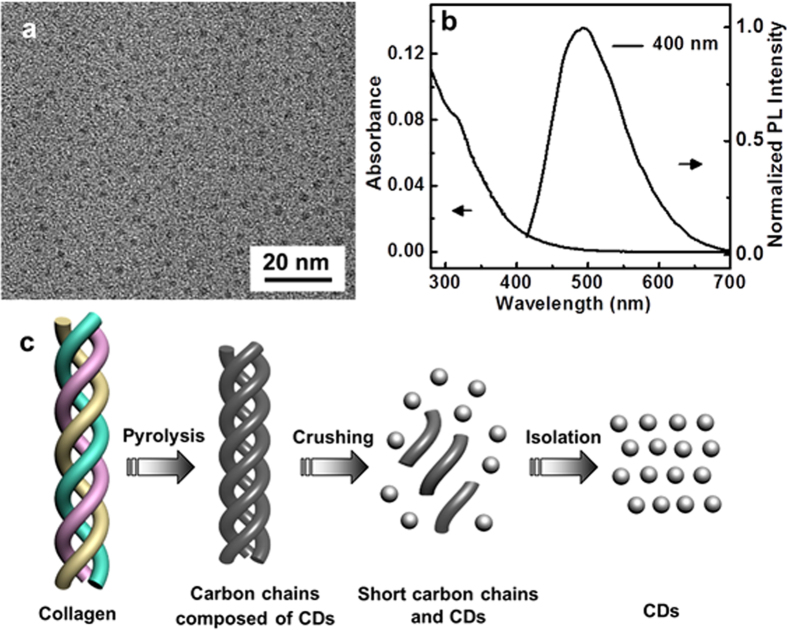
(**a**) HRTEM image of CDs. (**b**) UV-vis absorption and PL emission spectra of CDs in ethanol (*λ*_ex_ = 400 nm). (**c**) A possible mechanism for the generation of CDs from scales.

To investigate the PL mechanism of HCSs, the as-derived HCSs were crushed down to nanoscale and dispersed in ethanol. The resultant homogeneous supernatant contains strongly fluorescent CDs, which can be confirmed by the high-resolution transmission electron microscopy (HRTEM) and PL spectra (Fig. 5a,b). As shown in HRTEM image (Fig. 5a), these CDs appear as spherical particles, and have uniform dispersion without apparent aggregation and a mean diameter of 2.52 nm (Figure S6). No crystalline lattices were observed in the HRTEM image, which is consistent with the result of XRD characterization (Figure S4). Figure 5b and Figure S7 show optical properties of these CDs. An intense absorption characteristic peak at ~315 nm is observed in the UV-vis absorption spectrum. Excited at 400 nm, a PL peak centering at 495 nm with a full width at half maximum of about 110 nm is observed. Based on this UV-vis spectrum, the quantum confinement effect according to the band gap of the CDs was first investigated, and the CDs were considered as indirect band gap materials, as previously reported^34^. The indirect band gap *E*_g_ of CDs is 2.50 eV (Figure S8), which demonstrates a strong quantum confinement effect and shows the evidence of CDs with nanometer particle size. The quantum yields (QYs) of the as-prepared CDs was determined to be 38% (see details in Table S1), and this value is much better than those of most of CDs reported elsewhere^35^,^36^. It is worth noting that the scales exhibit blue fluorescence (Figure S9), which is quite different from the PL properties of CDs and HCSs. A possible mechanism for the generation of CDs from scales is illustrated in Fig. 5c. As a primary chemical component of scales, collagen has triple-helix structure with the supercoiling of three polypeptide chains^37^. High temperature offers powerful energy to break its chemical bonds, allowing long carbon chains to be decomposed to CDs during pyrolysis process. Therefore, the fluorescence of HCSs has no concern with the precursor but could be attributed to enriched CDs covering on their surface.

### Application of HCSs

The CDs derived from the HCSs were directly employed as inks for inkjet-printing multicolor patterns and sensitizers in solar cells. Figure S10 shows the multicolor PL patterns with different excitation wavelengths under the LCFM system. The butterfly pattern exhibits bright cyan, green and yellow color under 405 nm, 458 nm and 514 nm, respectively. By using this as-prepared CDs, the printing pattern can emit multicolor fluorescence without doping with other dyes^35^. The emission wavelengths of the CDs in solution are slightly blue-shifted in comparison with those of the HCSs. This phenomenon might be attributed to the energy transfer within an inhomogeneous distribution emitting species in the solid film^38^. The current density-voltage curve of a CD-sensitized solar cell (CDSSC) is shown in Figure S11. A short-circuit current density (*J*_sc_) of 0.65 mA cm^−2^ and an open-circuit voltage (*V*_oc_) of 0.55 V were observed with a fill factor (*FF*) of 78.8% and energy conversion efficiency (*η*) of 0.28%. These values are much better than the previously reported CD-sensitized solar cells (*J*_sc_ = 0.53 mA cm^−2^, *V*_oc_ = 0.38 V, *FF* = 64% and *η* = 0.13%)^39^ and graphene-dot-sensitized solar cells (*J*_sc_ = 0.2 mA cm^−2^, *V*_oc_ = 0.48 V and *FF* = 58%)^40^. The efficiency of the CDSSC is much lower than those of quantum dots sensitized solar cells. However, the CDs possess advantages of easy fabrication, lower cost, and lower toxicity compared to semiconductor QDs, and there is much space for further improvement. Therefore, the CDs derived from HCSs may have potential application in multicolor imaging and optoelectronic devices.

### Fabrication of HCSs on a large scale

**Figure 6:**
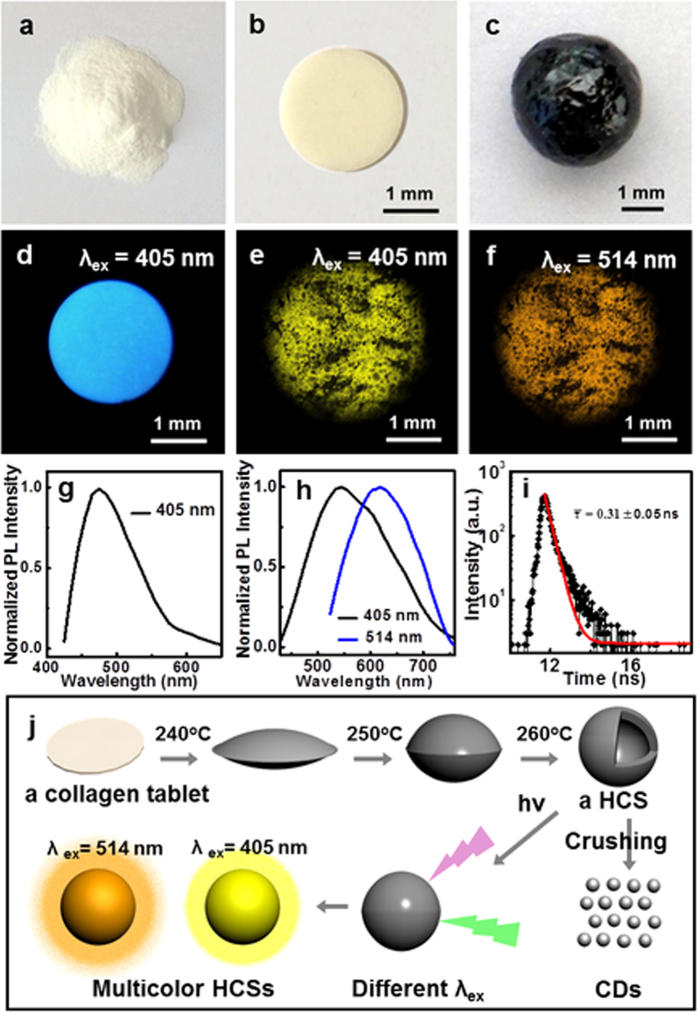
(**a–c**) Photographs of collagen powders, a collagen tablet and a HCS under ambient light. (**d**) Photograph of a collagen tablet under UV light (*λ*_ex_ = 405 nm). LCFM images of a HCS derived from collagen powders at different excitation wavelengths (**e**) 405 nm and (**f**) 514 nm. PL emission spectra of a collagen tablet (**g**) and a HCS (**h**) at different excitation wavelengths. (**i**) A typical time-resolved fluorescence decay curve of a HCS derived from collagen powders (*λ*_ex_ = 405 nm). (**j**) Schematic illustration of the formation process of a fluorescent HCS from a collagen tablet.

To realize large-scale production and non-pretreatment procedure for HCSs, we chose collagen powders as the precursor to replace fish scales. As reported, collagen is the primary chemical composition of scales, which could also be extracted from many other kinds of materials, such as skins, bones and shells. A collagen tablet with diameter about 2 mm was formed from collagen powders by compression molding (Fig. 6a,b), and exhibited blue fluorescence under UV excitation (Fig. 6d,g). Without pretreatment procedure, a tablet ballooned from its center gradually with similar process as scales, and an enclosed black sphere grew up with diameter of about 3 mm during the pyrolysis process, as depicted in Fig. 6c. The carbon sphere is also hollow but has rough surface. Confocal fluorescence micrographs of the HCS show that the yellow and orange luminescence emissions were observed with the excitation wavelength of 405 nm and 514 nm, respectively (Fig. 6e,f,h), which is a little red-shift in contrast with the PL of the HCSs derived from scales. This feature might be ascribed to higher concentration of CDs in the HCS derived from collagen powders than that from scales. The time-resolved photoluminescence measurement indicates that the decay lifetime of these HCSs is 0.31 ± 0.05 ns (Fig. 6i). The schematic illustration of the formation process of a fluorescent HCS is illustrated in Fig. 6j. Carbon dots could also be extracted from the collagen-derived HCSs, as depicted in Figure S12, which have the similar fluorescence to the CDs prepared from scale-derived HCSs. This investigation constitutes a simpler way to prepare fluorescent HCSs from collagen powders than from scales, which may open a promising avenue for the large-scale production of multicolor fluorescent HCSs.

In contrast to previous methods, this approach for fabrication of HCSs encapsulating enriched CDs offers the following advantages: (1) This method without any templates and post treating, is simple, environmentally friendly and versatile, and makes it attractive for preparing hollow carbon materials on an industrial scale. (2) The gas blow forming mechanism of HCSs here would help others to gain fundamental insight into other hollow materials. (3) The as-prepared HCSs confer unique multicolor fluorescence, which represent the first examples of multicolor fluorescent HCSs.

## Conclusions

In summary, we have developed a simple and versatile route for fabricating multicolor fluorescent HCSs with the use of natural scales and collagen powders as the precursor *via in situ* one-step pyrolysis process for the first time. The HCSs grow up by gas blow forming during the pyrolysis process and exhibit multicolor fluorescence while changing the excitation wavelength. PL emission mechanism of HCSs have been thoroughly discussed and prove that the HCSs encapsulate enriched CDs. The CDs broken off from the HCSs by top-down methods possess high QYs of 38%, and their applications in inkjet printing and sensitized solar cell have been also described. Therefore, this strategy might provide an available avenue to produce hollow carbon materials on a large scale with multicolor fluorescence performance, along with variety applications.

## Methods

### Preparation of fluorescent HCSs from scales and collagen powders

All the pretreatment of scales were at room temperature (25 ± 2 °C). To remove the fats and pigments on the surface, the whitefish scales (10.0 g) were soaked in 0.5 M Na_2_CO_3_ at a sample/alkaline solution ratio of 1:10 (w/v) for 24 h, changing the alkaline solution every 12 h. Later, alkaline solution was decanted and the scales were washed with water. Subsequently, the treated samples were decalcified with 0.6 M HCl at a sample/HCl solution ratio of 1:10 (w/v) for 3 days, with the HCl solution being changed every day. After being fully washed with cold distilled water, the pretreated scales were transparent and soft. The scales should be separated one by one and dried at room temperature. The dried sheets were stored in polythene covers for further research. HCSs were prepared by pyrolysis from pretreated scales. A quartz boat with three pretreated scales was transferred into a tube furnace, and was then pyrolyzed at 260 °C for 2 h under N_2_ flow with a heating rate of 5 °C/min. The three scales turned to be three black carbon spheres and were collected for further use and characterizations after cooled down to room temperature. Collagen powders (about 5 mg) was put in the tablet compression machine and pressed into a tablet with a diameter of 2 mm. The pressure we used is 10 MPa. Then a collagen tablet was transferred into a tube furnace and pyrolyzed with the same condition as scales. The as-derived HCSs were grinded in a mortar and dispersed in ethanol. The suspension solution was sonicated, followed by high-speed centrifugation (12000 rpm, 20 min) to remove large particles. The resultant homogeneous supernatant contained strongly fluorescent CDs.

### Characterization

Transmission electron microscope (TEM) observation was performed with a JEOL JEM-2100 transmission electron microscope. The morphology of the resulting samples was determined by scanning electron microscopy (SEM, QUANTA 200). Photographic images of the samples were captured by digital camera (100IS, Canon). Fluorescent images were recorded on the Leica TCS/SP5 laser confocal fluorescence microscopy (LCFM) system. Fluorescence decay time was measured based on the Leica TCS/SP5 fluorescence lifetime imaging microscopy (FLIM) system using a 405 nm laser as the excitation source. Thermogravimetric analysis (TGA) curves and the evolutionary FT-IR profiles of scales were collected on a NETZSCH STA 449 F3 Jupiter/Nicolet 6700 (TGA/FT-IR) system. The samples were combusted in the air at the temperature ranging from 40 °C to 500 °C (10 °C min^−1^). UV-vis absorption spectra were recorded by a UV-vis spectrometer (Lambda 950, Perkin-Elmer). Photoluminescence (PL) measurements were carried out on a Varian Cary Eclipse spectrophotometer. Elemental analysis of the HCS samples was performed on an Elementar Vario EL III. X-ray photoelectron spectroscopy (XPS) spectra of the CDs were performed on an ES-CAIAB250 XPS system with Al/K α as the source, and the energy step size was set as 1.000 eV.

## Additional Information

**How to cite this article**: Chen, Q.-L. *et al.* Direct Synthesis of Multicolor Fluorescent Hollow Carbon Spheres Encapsulating Enriched Carbon Dots. *Sci. Rep.* **6**, 19382; doi: 10.1038/srep19382 (2016).

## Supplementary Material

Supplementary Information
