# Supplementary material for: Direct Synthesis of Multicolor Fluorescent Hollow Carbon Spheres Encapsulating Enriched Carbon Dots
Source: Sci Rep. 2016 Jan 25;6:19382. doi: 10.1038/srep19382 (PMC4726404; doi:10.1038/srep19382)
Supplement: Supplementary Information [file srep19382-s1.doc]

**Supporting Information:**

**Direct Synthesis of Multicolor Fluorescent Hollow Carbon Spheres** **Encapsulating Enriched Carbon Dots**

Qiao-Ling Chen, Wen-Qing Ji, and Su Chen^*^

*State Key Laboratory of Materials-Oriented Chemical Engineering and College of Chemistry and Chemical Engineering, Nanjing Tech University, Nanjing 210009 (P. R. China)*

E-mail: [*chensu@njtech.edu.cn*](mailto:chensu@njtech.edu.cn)

**Experimental section**

**Materials**

Whitefish scales (0.3-0.5 kg) were purchased from a fish market in Nanjing City, China. They were washed throughly in distilled water and dried at 60 ^o^C in a hot air oven for further use. Collagen powders was purchased from Zhenya Chemical Co., Ltd. (China) with a purity of 99 percent. Reagent-grade toluene, ethanol, acetone, cyclohexane, dichloromethane (CH_2_Cl_2_), sodium carbonate (Na_2_CO_3_) and hydrochloric acid (HCl) were purchased from standard sources. Fluorine doped tin oxide electrodes with a 6 mm × 6 mm square TiO_2_ film and (I_3_^-^/I^-^) acetonitrile electrolyte (DHS-E23) were purchased from the Dalian Rainbow Solar Technology Development Co., Ltd. (DaLian, China). All chemicals were used as received. High-purity water with the resistivity of greater than 18 MΩ cm^-1^ was used in the experiments.

**Patterning from the CDs solution**

For inkjet printing, 10 g ethanol solution of CDs (2 wt %) was transferred to the chamber of the Jetlab®II Precision Printing Platform at room temperature. And a predefined picture was used in the printing program. In our case, a 10 mm×13 mm butterfly pattern was printed on a filter paper.

**Fabrication of solar cells**

The CDs/TiO_2_ FTO electrode was prepared by immersing TiO_2_-FTO electrode in the CDs ethanol solution for 48 h in the dark. The CDs /TiO_2_-FTO electrode was finally dried under vacuum. The CDs adsorbed TiO_2_ photoanode and Pt counter electrode were assembled into a sandwich type cell and sealed with a hot-melt gasket of 25mm thickness made of theionomer Surlyn 1702 (Dupont). The electrolyte solution (acetonitrile solution containing I^-^/ I_3_^-^ redox couple, Rainbow Solar Technology) was infiltrated into the cell through a drilled hole in the counter electrode.

**Characterization**

Fourier-transform infrared (FT-IR) spectra were recorded on a Nicolet 6700 FT-IR spectrometer. Raman spectra were performed using a Horiba HR 800 Raman system equipped with a 514.5 nm laser. X-ray diffraction (XRD) was performed on a SmartLab of Rigaku Corporation. Inkjet printing was achieved base on the Jetlab®II Precision Printing Platform. The diameter of the micropipette nozzle of the printer is 60 μm, and the positioning accuracy of the print head is 1μm. The signal that drives the actuator is a rectangular pulse, with amplitude of 70 V and the ejection frequency of 800 Hz. The photocurrent-voltage experiments were performed under AM 1.5G solar simulator (Oriel, USA) as light source with an illumination intensity of 100 mW cm^-2^. The active area of the cell was typically 0.36 cm^2^. The current density-voltage (*J*-*V*) response of the devices was recorded using an electrochemical workstation (CHI 660C Instruments). All the spectra were measured at room temperature.


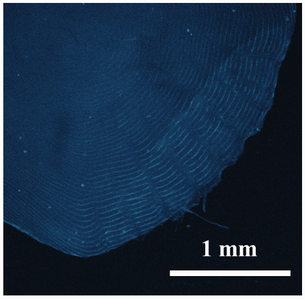


Figure S1. Confocal fluorescence microscopy image of a scale (*λ*_ex_ = 405 nm).


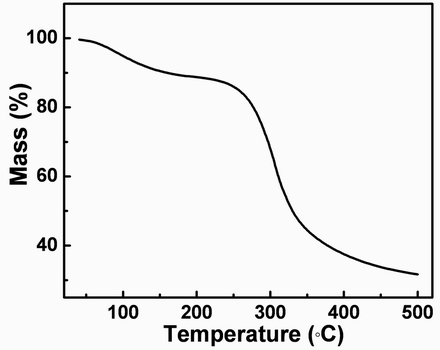


Figure S2. TGA curve of scales from 40 ^o^C to 500 ^o^C.


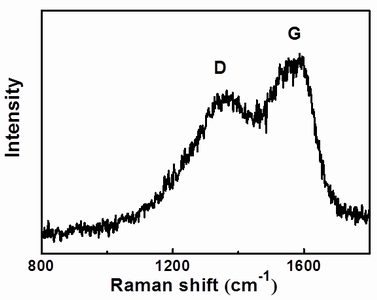


Figure S3. Raman spectrum of HCSs.


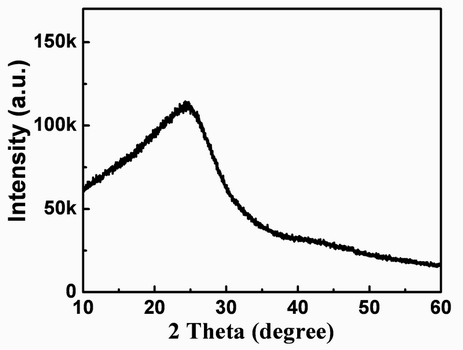


Figure S4. XRD pattern of HCSs.


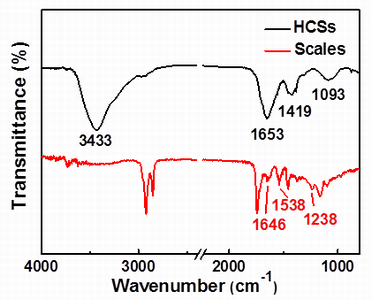


Figure S5. FT-IR spectra of HCSs and scales.


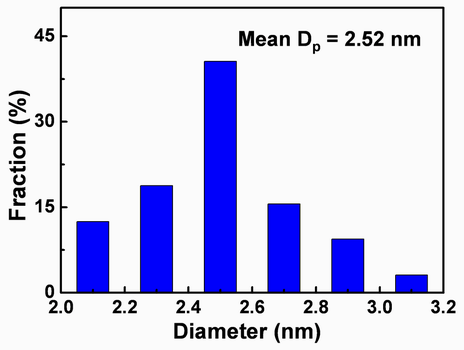


Figure S6. Particle size distribution of CDs.


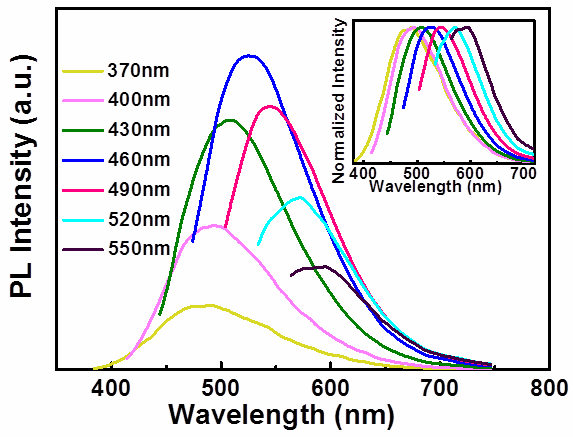


**Figure S7.** PL emission spectra of CDs in ethanol at different excitation wavelengths. Inset: normalized PL emission spectra.


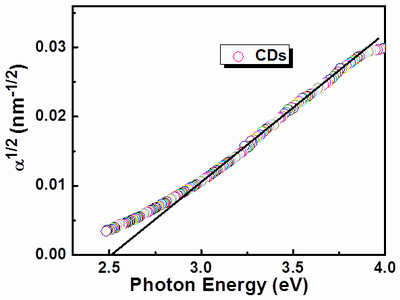


Figure S8. Plot of *α*^1/2^ vs *E*_phot_ for indirect transitions, where *α* is absorption coefficient and *E*_phot_ is photon energy. Band gaps *E*_g_ are obtained by extrapolation to *α* =0.

Based on the UV-vis spectrum, the quantum confinement effect of the CDs was investigated according to the band gaps. The method is as reported in literature.^1^ Figure 5b depicts UV-vis absorption spectra of CDs in ethanol. The data at wavelengths less than 290 nm are omitted due to the dispersive medium effect. The concentration of the CDs solution *c* = 7.6×10^-6^ g dm^-3^, which corresponds to an effective thickness *d* = 94 nm by the following formula: *d* = (*lc*/*ρ*), where *l* is the present path length, 10 mm, and *ρ* is the CDs density, 8.05×10^-4^ g mm^-3^. Figure S8 shows Plot of *α*^1/2^ vs *E*_phot_ for indirect transitions, where the absorption coefficient *α* = *A*/*d* and photon energy *E*_phot_ =1239/*λ* (eV) with *λ* the wavelength in nanometers. The value of *E*_phot_ extrapolated to *α* = 0 gives an absorption edge energy which corresponds to a band gap *E*_g_. As can be seen from Figure S8, the indirect band gap is 2.50 ev.

Table S1. Quantum yield of the as-prepared CDs.

| Sample | Integrated Emission Intensity | Abs@370nm (A) | Refractive index of solvent (η) | Quantum Yield (Q) |
| --- | --- | --- | --- | --- |
| Quinine sulfate | 7082.2 | 0.0248 | 1.33 | 0.54 (known) |
| CDs in ethanol | 3340.5 | 0.0309 | 1.362 | 0.38 |


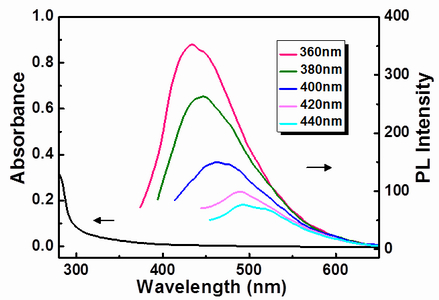


Figure S9. UV-vis absorption and PL emission spectra of scales.


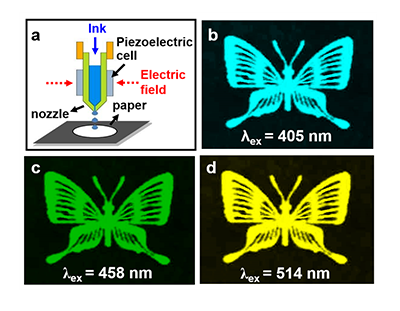


Figure S10. a) Scheme of piezoelectric printing. b-d) the PL patterns under different excitation of 405 nm, 458 nm and 514 nm in the LCFM system.


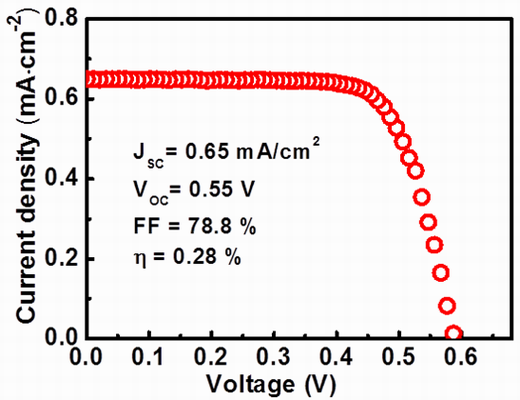


Figure S11. Current density-voltage (*J*-*V*) characteristics of CDs sensitized solar cell.


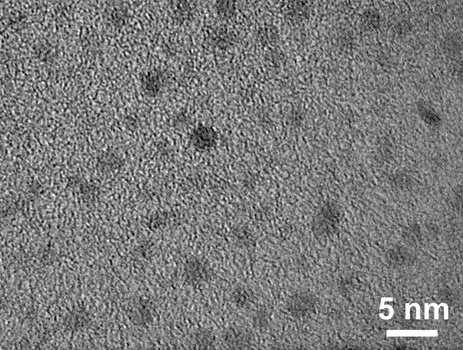


Figure S12. TEM of carbon dots derived from collagen powders.

**Reference**

1. Tsunekawa, S. & Fukuda, T. Blue shift in ultraviolet absorption spectra of monodisperse CeO_2-x_ nanoparticles.  *J. Appl. Phys.* **87**, 1318-1321 (2000).
